# Predicting mortality in non-cystic fibrosis bronchiectasis patients using distance-saturation product

**DOI:** 10.1080/07853890.2021.1999490

**Published:** 2021-11-11

**Authors:** Chun-Yu Lin, Meng-heng Hsieh, Yueh-Fu Fang, Chien-Wei Peng, Jia-Shiuan Ju, Yu-Lun Lo, Shu-Min Lin, Horng-Chyuan Lin

**Affiliations:** a Department of Thoracic Medicine, Chang Gung Memorial Hospital at Linkou, Taoyuan, Taiwan; b College of Medicine Chang Gung University, Taoyuan, Taiwan; c Department of Gastroenterology and Hepatology, Chang Gung Memorial Hospital at Linkou, Taoyuan, Taiwan; d Department of Respiratory Therapy, Chang Gung Memorial Hospital at Linkou, Taoyuan, Taiwan

**Keywords:** Mortality, non-cystic fibrosis bronchiectasis, bronchiectasis severity index, FACED, distance-saturation product

## Abstract

**Background:**

The bronchiectasis severity index (BSI) and FACED score are currently used in predicting outcomes of non-cystic fibrosis bronchiectasis (NCFB). Distance-saturation product (DSP), the product of distance walked, and lowest oxygen saturation during the 6-min walk test showed strong predictive power of mortality in non-CF bronchiectasis patients. This study aimed to compare the efficacy of these scores and DSP in predicting mortality.

**Methods and Patients:**

Our retrospective study included NCFB patients from January 2004 to December 2017. We recorded the basic data, pulmonary function, radiologic studies, sputum culture results, acute exacerbations (AE), emergency department (ED) visits, hospitalization, and mortality.

**Results:**

A total 130 NCFB patients were analysed. The mean BSI score, FACED score, and DSP were 8.8 ± 4.9, 3.4 ± 1.7, and 413.1 ± 101.5 m%, respectively. BSI and FACED scores had comparable predictive power for AE (*p*=.011; *p*=.010, respectively). The BSI score demonstrated a significant correlation with ED visits (*p*=.0003). There were 12 deaths. Patients were stratified using a DSP cut-off value of 345 m% according to the best area under receiver operator characteristic curve (AUC) value in mortality. DSP was not correlated with AE and ED visits. BSI, FACED scores, and DSP demonstrated statistically significant correlations with hospitalization (*p*<.0001; *p*<.0001; *p*=.0007, respectively). The AUC for overall mortality was similar for BSI, FACED score, and DSP (0.80 versus 0.85, *p*=.491; 0.85 versus 0.83, *p*=.831).

**Conclusion:**

DSP had comparable predictive power for mortality as the well-validated BSI and FACED scores and is relatively easy to use in clinical practice.

**KEY MESSAGE**
- Distance-saturation product (DSP) comprised with the product of distance walked, and lowest oxygen saturation during the 6-min walk test, which is common used in clinical practice.
- DSP demonstrated strong and comparable predictive power of mortality as the well-validated BSI and FACED scores in non-CF bronchiectasis patients.

## Introduction

Non-cystic fibrosis bronchiectasis (NCFB) is a progressively inflammatory, destructive lung disease, characterised by mucus impaction, bronchial dilatation, and recurrent infections [1–3]. There are two validated severity scores for NCFB: the bronchiectasis severity index (BSI) and FACED score [4,5]. Both of them consist of forced expiratory volume in 1 s (FEV_1_), age, chronic colonization of *Pseudomonas aeruginosa*, radiological extension, and dyspnoea [4,5]. As McDonnell et al. reported, the BSI score appears to better predict hospital admission, exacerbation, quality of life, and lung function decline than the FACED score [6]. This may attribute to the addition of body mass index (BMI), colonization with other microorganisms, hospitalization and exacerbation frequency in BSI score. Nevertheless, in a 19-year cohort, Ellis et al. demonstrated that the FACED score had slightly superior predictive power for 15-year mortality [7]. The BSI and FACED scores are both useful in predicting outcomes of non-CF bronchiectasis, but the complexity of BSI scores limits the use in daily practice.

The 6-min walk test (6MWT) is a useful exercise test to evaluate cardiovascular and pulmonary functional status in many diseases [8–11]. Distance-saturation product (DSP) is the product of distance walked and lowest oxygen saturation during the 6MWT, which is correlated with survival in idiopathic pulmonary fibrosis (IPF) patients [12]. We previously conducted a retrospective study comparing each parameter collected from the 6MWT and showed that DSP had strong predicting power for mortality in NCFB patients [13]. In the Asian context, only Wang et al. conducted a retrospective study with validated BSI and FACED scores involving post-tuberculosis bronchiectasis patients [14]. This study aims to compare the prediction power of BSI, FACED score, and DSP for mortality in NCFB patients.

## Methods

### Subjects

This retrospective study was conducted from January 2004 to December 2017 at the Linkou Chang Gung Memorial Hospital, Taiwan. All adult patients underwent high-resolution computed tomography, confirming bronchiectasis. Patients were excluded if they had active tuberculosis, cystic fibrosis or malignancy, not performed 6MWT or who was under O2 supplement.

We recorded the following demographic characteristics: age, sex, body mass index (BMI), sputum culture results, pulmonary function, radiographic reports, medications, exacerbations, and mortality. Data for calculating the BSI and FACED scores were taken from the patients’ medical records. For every patient, we cultured at least two sputum samples for *Pseudomonas spp*. Colonization with *P. aeruginosa* was defined as ‘the isolation of *P. aeruginosa* in sputum culture on two or more occasions, at least 3 months apart in a 1-year period’. As we reported previously [15], we defined acute exacerbation (AE) as an event that was clinically diagnosed by the physician and required antibiotic prescription for acute onset of increasing cough, worsening dyspnoea, and changes in sputum characteristics (e.g. volume, consistency, and purulence). We also recorded the frequency of ED visits and hospitalization because of bronchiectasis AE in 2 years. All-cause mortality was ascertained as of December 2017 and dates of death obtained from medical records.

The study was approved by the institutional review board of Chang Gung Memorial Hospital (approval no. 201802305A3). Informed consent was waived because this was a retrospective study and there was no modification in patient management. All personal information was encrypted in a database, and patient data were anonymized. There was no breach of privacy.

### 6mwt and pulmonary function tests

The 6MWT was performed in accordance with the standard protocol of the American Thoracic Society [11,16] under the supervision of well-trained technicians. All 6MWT were performed with pulse oximetry for continuous recording of oxygen saturation (SpO2). Dyspnoea was assessed using the Borg scale each minute during the 6MWT and maximum dyspnoea level was recorded [17]. Spirometry was performed as per recommendations made by the American Thoracic Society/European Respiratory Society [18]. We excluded 6MWTs performed under oxygen supplement, and all 6MWTs were performed in room air. Variables from 6MWT, including 6-min walk distance (6MWD), and serial oxygen saturation in exercise were recorded. DSP was defined as the product of the final 6MWD in metres and the lowest oxygen saturation in room air during the 6MWT [12,13]. For example, a patient walking a total of 200 m with the lowest oxygen saturation of 88% during 6MWT would have a DSP of 176 m% (e.g. 200 × 0.88).

### Categorization

In addition to the calculation of severity scores, disease status also categorised as mild (BSI score ≤4, FACED score ≥2), moderate (BSI score 5–8, FACED score 3–4), and severe (BSI score ≥9, FACED score ≥5) disease according to each scoring system. The patients were stratified using a DSP cut-off value of 345 m% into two groups according to the best area under the curve (AUC) value in predicting mortality.

### Statistical analyses

We used Fisher’s exact test to compare categorical variables and were described using counts (percentages). Parametric data were expressed as means ± standard deviations. Mean differences were compared using the *t*-test (or analysis of variance for more than two groups). Non-parametric data were compared using Chi-square test. The receiver operating characteristic (ROC) curves were calculated, and AUC compared using DeLong’s test for two correlated ROC curves, where appropriate, was used to determine the statistical significance of the difference between the means. Optimum threshold values were identified as those yielding the highest Youden’s index (sensitivity + [1 − specificity]) [7]. After identifying the DSP that generated the highest AUC, actuarial survival curves were then constructed for comparison of DSPs above this threshold to those falling below this point. Survival analysis was performed using the Kaplan–Meier method. Kaplan–Meier curves were compared using the log-rank test. In further analyses, the participants were stratified into two groups according to the best AUC measurement using a DSP cut-off value of 345 m%. All analyses were two-sided, and *p*<.05 was considered statistically significant. Statistical analyses were performed using Prism version 5 (GraphPad Software Inc., La Jolla, CA, USA).

## Results

### Patients’ characteristics

**Figure 1.**
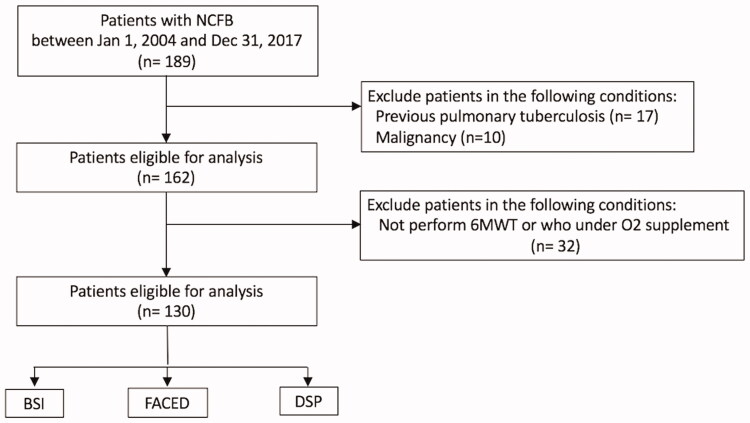
Flowchart of the study design. BSI: bronchiectasis severity index; DSP: distance-saturation product; NCFB: non-cystic fibrosis bronchiectasis; O2: Oxygen; 6MWT: 6-minute walk test.

**Figure 2.**
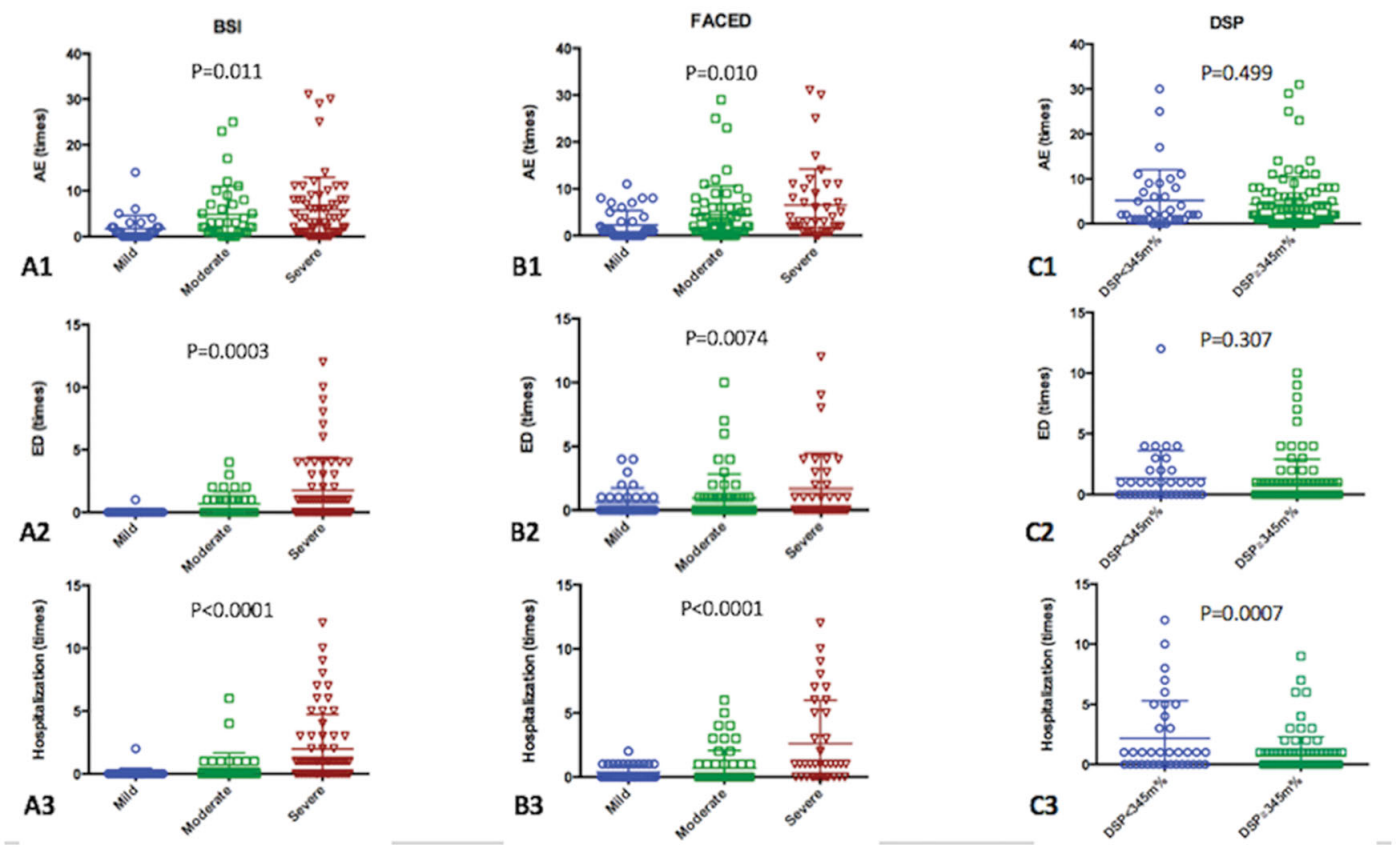
Comparison of AE, ED visit, Hospitalization using BSI score, FACED score and DSP. Notes: (A) Classified patients to mild, moderate and severe group by BSI score; (B) Classified patients to mild, moderate and severe group by FACED score; (C) Classified patients using a DSP cut-off value of 345 m%. AE: acute exacerbations; ED: emergency department; BSI: bronchiectasis severity index; DSP: distance-saturation product.

Data from all 130 adult patients diagnosed with NCFB were analyzed (Figure 1). The median duration of follow-up was 80 months (range, 24–216 months). Patient demographics are shown in Table 1. The mean age of the patients was 66.5 ± 11.9 years. The mean BSI score, FACED score, and DSP were 8.8 ± 4.9, 3.4 ± 1.7, and 413.1 ± 101.5 m% (Table 1). The BSI score categorised 49% of the patients as severe compared with 28% categorised by the FACED score (Table 2). While BSI and FACED scores had comparable predictions for AE (*p*=.011; *p*=.010, respectively, Table 2), BSI showed more significant correlation with ED visit than FACED score (*p*=.0003; *p*=.074, respectively, Table 2). The study patients were further stratified using a DSP cut-off value of 345 m% into two groups according to the best AUC value in mortality. Two, eight, and 26 patients had mild, moderate, and severe BSI, respectively, as classified by DSP of <345 m%. Two, 11, and 23 patients with mild, moderate, and severe FACED scores, respectively, had DSP <345 m%. DSP was not correlated with AE and ED visit. Nevertheless, BSI, FACED scores, and DSP all demonstrated statistically significant correlations with hospitalization (*p*<.0001; *p*<.0001; *p*=.0007, respectively, Table 2, Figure 2).

**Table 1.** Clinical characteristics of NCFB patients.

Variables	**All patients** *n* = 130
Age (years), mean ± SD	66.5 ± 11.9
Male, *n* (%)	57 (44)
BMI (kg/m^2^), mean ± SD	22.7 ± 3.3
Comorbidity	
Hypertension	15 (12)
Ischemic heart disease	3 (2)
Diabetes mellitus	11 (8)
Solid tumours	3 (2)
Liver diseases	4 (3)
Chronic kidney disease	1 (1)
FEV1, %predicted, mean ± SD	62.3 ± 22.9
*Pseudomonas* colonization, *n* (%)	39 (30)
mMRC dyspnoea score, mean ± SD	1.9 ± 0.9
Lobes affected, mean ± SD	2.8 ± 1.1
Hospitalizations in last 2 years, mean ± SD	1.1 ± 1.8
Exacerbations in previous year, mean ± SD	1.3 ± 2.1
BSI, mean ± SD	8.8 ± 4.9
FACED, mean ± SD	3.4 ± 1.7
DSP, m%, mean ± SD	413.1 ± 101.5
Death, *n* (%)	12 (9)

SD: standard deviation; BMI: body mass index; mMRC: modified Medical Research Council; BSI: bronchiectasis severity index; DSP: distance-saturation product.

**Table 2.** Comparison of exacerbation frequency and severity in patients with NCFB according to the BSI, FACED scores, and DSP.

	BSI				FACED				DSP		
	Mild *n* = 29	Moderate *n* = 37	Severe *n* = 64	p value	Mild *n* = 36	Moderate *n* = 57	Severe *n* = 37	p value	<345 *n* = 36	≥345 *n* = 94	p value
AE	1.7 ± 2.9	4.8 ± 6.1	5.9 ± 7.1	.011	2.3 ± 3.1	4.5 ± 6.1	6.6 ± 7.7	.010	5.2 ± 1.1	4.4 ± 0.6	.499
ED visit	0.03 ± 0.2	0.7 ± 1.0	1.8 ± 2.7	.0003	0.6 ± 1.1	0.9 ± 1.9	1.7 ± 2.8	.074	1.4 ± 0.4	0.9 ± 0.2	.307
Hospitalization	0.07 ± 0.4	0.5 ± 1.2	2.0 ± 2.8	<.0001	0.4 ± 0.5	0.7 ± 1.4	2.6 ± 3.4	<.0001	2.2 ± 0.5	0.7 ± 0.2	.0007

BSI: bronchiectasis severity index; DSP: distance-saturation product; AE: acute exacerbation; ED: emergency department.

### Survival analysis

Of the 130 patients, 12 died (9%) during the study period. Mortality varied according to BSI and FACED scores, from 3% and 0% in mild cases to 16% and 27% for those with severe scores. Using a DSP cut-off value of 345 m%, the mortality was 3% in those with high DSP and was 25% in those with worse DSP (*p*<.0001, Table 3).

**Table 3.** Univariate Cox proportional hazard analysis of BSI, FACED scores, and DSP for mortality during the study period.

	Subjects, *n*	Mortality, *n* (%)	Hazard ratio (95% CI)	p value
**BSI**				
Mild	29	1 (3)	Reference	–
Moderate	37	1 (3)	0.88 (0.05–14.14)	.928
Severe	64	10 (16)	6.08 (1.03–11.82)	.047
**FACED**				
Mild	36	0 (0)	Reference	–
Moderate	57	3 (5)	4.05 (0.17–95.39)	.385
Severe	37	10 (27)	5.38 (1.43–20.22)	.0128
**DSP**				
≥345	94	3 (3)	Reference	–
<345	36	9 (25)	9.03 (4.02–54.96)	<.0001

BSI: bronchiectasis severity index; DSP: distance-saturation product.

**Figure 3.**
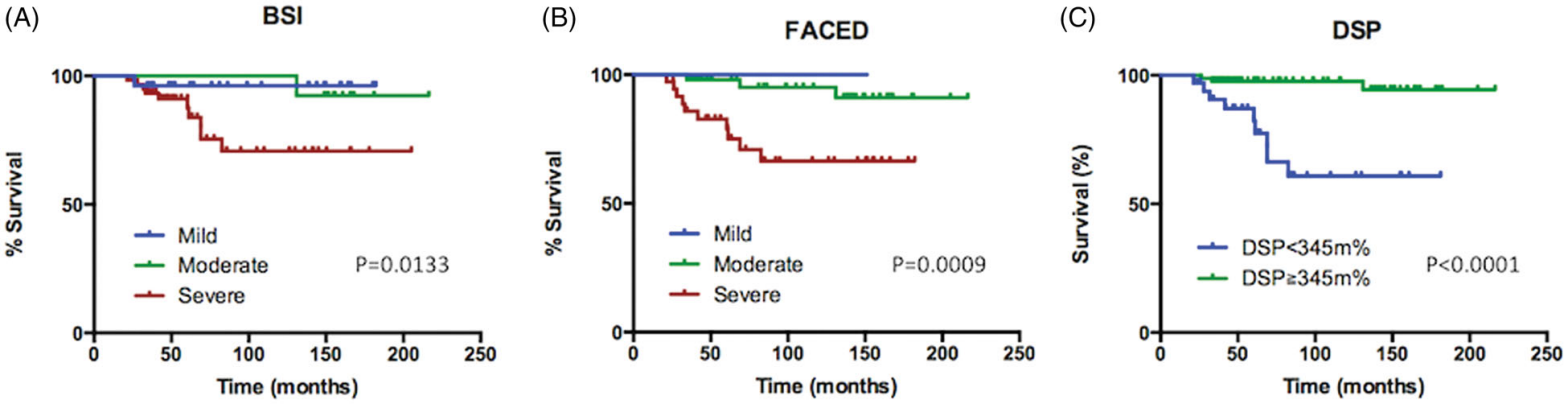
Survival of patients with non-cystic fibrosis bronchiectasis. (A) Survival in relation to BSI score, (B) Survival in relation to FACED score, (C) Survival in relation to DSP. BSI: bronchiectasis severity index; DSP: distance-saturation product.

Kaplan–Meier survival curves for mortality according to BSI, FACED scores, and DSP are shown in Figure 3. There was little difference between mild and moderate BSI scores (*p*=.928). Severe BSI score had significantly reduced the survival rate (hazard ratio: 6.08, *p*=.047). Regarding the FACED score, mild and moderate cases had no survival difference (*p*=.385). The severe group had significantly reduced survival compared with the mild group (Hazard ratio:5.38, *p* = 0.0128). Patients who had worse DSP (<345 m%) demonstrated lower death rate than those with high DSP (≥345 m%) (hazard ratio:9.03, *p*<.0001). The results of univariate Cox proportional hazards analysis are shown in Table 3.

### Receiver operating characteristic analysis

**Figure 4.**
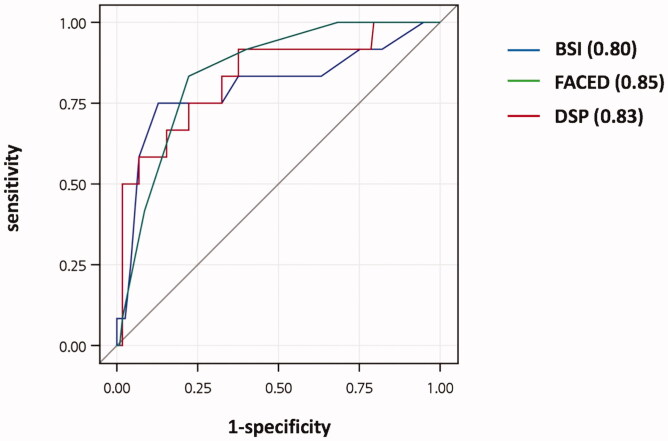
ROC curves of survival in patients with non-cystic fibrosis bronchiectasis. ROC: receiver operating characteristic; BSI: bronchiectasis severity index; DSP: distance-saturation product.

The AUC for overall mortality was similar for BSI, FACED scores, and DSP (0.80 versus 0.85, *p*=.491; 0.85 versus 0.83, *p*=.831, Table 4, Figure 4). The optimum threshold of >9.5 for the BSI score yielded a specificity and sensitivity of 61.9% and 83.3%, respectively; for the FACED score, a threshold of >4.5 yielded a specificity and sensitivity of 78.0% and 83.3%, respectively; for the DSP, a threshold of ≥345 m% yielded a specificity and sensitivity of 77.1% and 75%, respectively.

**Table 4.** Area under the curve (AUC) for mortality at different time points between BSI, FACED scores, and DSP.

	BSI	FACED	DSP
**Mortality**	0.80 (0.63–0.96)	0.85 (0.75–0.94)	0.83 (0.70–0.96)
	^#^*p*=.491	^@^*p*=.831	*p=.578

Data are presented as area under the curve (95% CI).

p-values calculated using DeLong’s test for two correlated ROC curves.

^#^Comparison of ROC curves between BSI and FACED; ^@^comparison of ROC curves between FACED and DSP; *comparison of ROC curves between DSP and BSI.

BSI: bronchiectasis severity index; DSP: distance-saturation product.

## Discussion

To the best of our knowledge, this is the first study to evaluate the prognostic value of DSP and compare it with that of the validated BSI and FACED scores in NCFB patients. In the 130 patients with a median follow-up duration of 80 months, both BSI and FACED scores were positively correlated with AE and ED visit and DSP was not. Nevertheless, similar to BSI and FACED score, DSP was strongly associated with hospitalization. Moreover, in the ROC analysis, BSI, FACED score, and DSP had comparable AUCs in predicting mortality.

The BSI and FACED scores are two well-known prognostic indices for bronchiectasis [4,5]. Both comprise age, value of FEV1% predicted, chronic colonization by *P. aeruginosa*, radiological extension of bronchiectasis, and degree of dyspnoea. BSI also considers BMI, exacerbation frequency, prior hospitalization for exacerbation, and chronic colonization with bacteria other than *P. aeruginosa*. Both scores classify patients into low-, moderate-, and high-risk groups, using different thresholds. In the UK, Ellis et al. analysed 74 patients with a median follow-up of 18.8 years and demonstrated that the AUC for the 5-year mortality was similar for BSI and FACED scores, although the FACED score was superior to BSI in predicting 15-year mortality [7]. McDonnell et al. conducted an analysis of seven European cohorts when comparing the BSI and FACED score. They found that BSI predicted hospital admissions, exacerbations, quality of life, and lung function decline precisely while both BSI and FACED scores had comparable predicting power for mortality [6]. BSI score appears to have better prediction in hospital admission, exacerbation than the FACED score, this may attribute to the addition of hospitalization and exacerbation frequency in BSI score. Only one retrospective study in Asia, conducted by Wang et al. in China showed that the AUC of the FACED score in predicting 4-year mortality was 0.81 and that of the BSI was 0.70 in post-tuberculosis bronchiectasis patients [14]. In the current research, we found that both BSI and FACED scores were significantly correlated with acute exacerbation, hospitalization, and mortality. The BSI score showed a more significant association with ED visit than the FACED score. Because of clinic visit and hospitalization are relative accessible in Taiwan. This may have overestimated the BSI score and led to some bias.

The 6MWT is a convenient clinical evaluation tool and many variables derived from the 6MWT are useful for evaluating prognosis, including hospitalization and mortality [19,20]. The DSP is a composite measure that reflects both exercise capacity and desaturation and was first introduced in predicting mortality in IPF patients [12]. Recently, Huang et al. showed that exertional desaturation during the 6MWT was a predictive factor for osteoporosis in NCFB patients [21]. In our previous research, we found that DSP predicted 6-year mortality in NCFB [13]. In the current analysis, we compared the BSI and FACED score with DSP in predicting mortality and demonstrated comparative results. The AUCs of overall mortality were 0.8 for BSI, 0.85 for FACED score, and 0.83 for DSP, similar to the previously reported AUCs of BSI and FACED score in other large study cohorts [6,7,22].

As mentioned above, the BSI played a complimentary role to the FACED score in predicting the exacerbation and hospitalization risk [23]. For better prediction for exacerbations in clinical practice, Mayor et al. proposed a modified version of FACED by adding previous exacerbations (Exa-FACED) and found that it could improve the AUC for exacerbations and hospitalizations [22]. Carrillo et al. further validated the Exa-FACED score in predicting all-cause mortality [24]. In the current study, both the BSI and FACED score were closely correlated with AE and admission. The BSI was associated with ED visit, although the FACED score was not. The DSP was only correlated with hospitalization and not AE and ED visit. This may be attributed to the fact that the DSP does not include previous exacerbation. However, with regard to mortality, the DSP demonstrated comparable predicting power as the BSI and FACED score. Introduction of the DSP in the context of these well-validate scores may increase the discriminatory power of BSI or FACED scores. Further large-scale, prospective studies are required.

Our study has some inherent limitations. First, it was a retrospective study, and we did not record quality of life (QoL) in the chart, therefore, we did not provide information about QoL. Second, DSP did not include previous AE and may have diminished the predicting power of AE, ED visit, and hospitalization. Moreover, clinic visit, ED visit, and hospitalization are relative accessible in Taiwan. This may have overestimated the BSI score and led to some bias. Fourth, our hospital was tertiary medical centre, the patients might have features of more severe bronchiectasis. Fifth, we couldn’t approach patients who look for another hospital while they developed AE. To validate the usefulness of the BSI score, FACED score, and DSP, cross-country, multicentre, large-scale, prospective studies are required.

## Conclusions

DSP had comparable predictive power for mortality as the validated BSI and FACED scores and is relatively easy to use. Further cross-country studies are warranted to validate the usefulness and calculate the cut-off value of DSP in predicting mortality in NCFB patients.

## Data Availability

The data sets analysed during the current study are available from the corresponding author upon reasonable request.

## Abbreviations

BSI: bronchiectasis severity index
NCFB: non-cystic fibrosis bronchiectasis
DSP: distance-saturation product
AE: acute exacerbations
ED: emergency department
FEV_1_: forced expiratory volume in one second
6MWT: 6-minute walk test
IPF: idiopathic pulmonary fibrosis
FVC: forced vital capacity
BMI: body mass index
6MWD: 6-minute walk distance
AUC: area under the curve
ROC: receiver operating characteristic
QoL: quality of life
